# Contribution of the inflammasome to inflammaging

**DOI:** 10.1186/s12950-018-0198-3

**Published:** 2018-11-16

**Authors:** Nancy H. Mejias, Camila C. Martinez, Marisa E. Stephens, Juan Pablo de Rivero Vaccari

**Affiliations:** Department of Neurological Surgery, Lois Pope LIFE Center, The Miami Project to Cure Paralysis, Miller School of Medicine, University of Miami, 1095 NW 14th Terrace, 3-25, Miami, FL 33136-1060 USA

**Keywords:** Inflammasome, Aging, Oxidative stress, Caspase-1, ASC, Inflammation, Inflammaging

## Abstract

**Background:**

Inflammation is a natural part of the aging process. This process is referred to as inflammaging. Inflammaging has been associated with deleterious outcomes in the aging brain in diseases such as Alzheimer’s disease and Parkinson’s disease. The inflammasome is a multi-protein complex of the innate immune response involved in the activation of caspase-1 and the processing of the inflammatory cytokines interleukin (IL)-1β and IL-18. We have previously shown that the inflammasome plays a role in the aging process in the brain. In this study, we analyzed the brain of young (3 months old) and aged (18 months old) mice for the expression of inflammasome proteins.

**Results:**

Our findings indicate that the inflammasome proteins NLRC4, caspase-1, apoptosis-associated speck-like protein containing a caspase recruitment domain (ASC), and IL-18 are elevated in the cytosol of cortical lysates in aged mice when compared to young. In addition, in the cytosolic fraction of hippocampal lysates in aged mice, we found an increase in NLRC4, caspase-1, caspase-11, ASC and IL-1β. Moreover, we found higher levels of ASC in the mitochondrial fraction of aged mice when compared to young, consistent with higher levels of the substrate of pyroptosis gasdermin-D (GSDM-D) and increased pyroptosome formation (ASC oligomerization). Importantly, in this study we obtained fibroblasts from a subject that donated his cells at three different ages (49, 52 and 64 years old (y/o)) and found that the protein levels of caspase-1 and ASC were higher at 64 than at 52 y/o. In addition, the 52 y/o cells were more susceptible to oxidative stress as determined by lactose dehydrogenase (LDH) release levels. However, this response was ameliorated by inhibition of the inflammasome with Ac-Tyr-Val-Ala-Asp-Chloromethylketone (Ac-YVAD-CMK). In addition, we found that the protein levels of ASC and IL-18 are elevated in the serum of subjects over the age of 45 y/o when compared to younger subjects, and that ASC was higher in Caucasians than Blacks and Hispanics, whereas IL-18 was higher in Caucasians than in blacks, regardless of age.

**Conclusions:**

Taken together, our data indicate that the inflammasome contributes to inflammaging and that the inflammasome-mediated cell death mechanism of pyroptosis contributes to cell demise in the aging brain.

## Background

Aging is a complex phenomenon associated with environmental, stochastic, genetic and epigenetic factors affecting all tissues of the body over time. Aging of the brain is a common-denominator in several neurodegenerative diseases [1]. A factor associated with aging is cognitive decline. Cognitive decline is highly conserved among mammals, including humans, rodents, monkeys and dogs [2–4]. Associated with the process of aging is chronic inflammation. Inflammaging, or aging-related inflammation, is a risk factor for morbidity and mortality in the elderly population, and it is regulated in part by the innate immune response. Therefore, targeting the inflammatory response in the aging brain has the potential to improve cognitive performance. In this regard, we have provided the first evidence of inflammasome activation in the hippocampus of aged rats. Accordingly, rats that were treated with probenecid, a non-specific inflammasome inhibitor, showed decreased activation of the inflammasome [4]. Importantly, this effect was associated with improved spatial learning performance [4]. Thus, modulation of inflammation in the brain is a promising approach to improving cognitive performance in the elderly population.

As a result, there is a need for a greater understanding of the innate immune response in the aging population. In this project, we studied the brain of young (3 months) and aged (18 months) mice at two different ages to identify the cytosolic and mitochondrial distribution of inflammasome signaling proteins in the cortex and hippocampus. Importantly, since oxidative stress is a main contributor to the aging process [5], we also studied the effects of oxidative stress on aging by exposing fibroblasts from a subject who donated his cells at three different ages (49, 52 and 64 years of age) to hydrogen peroxide and then determining the levels of lactose dehydrogenase (LDH) released and formation of cellular reactive oxygen species (ROS) after inflammasome inhibition.

In this study, we extent our previous knowledge on the role of the inflammasome in aging by showing that the inflammasome contributes to the oxidative stress and cell damage that is present as a result of aging. In addition, here we present a case in which inflammasome protein expression was increased in the fibroblasts from one individual who donated his cells at three different ages. Moreover, this is the first report to show the normal levels of inflammasome signaling proteins in young and middle aged individuals as well as in Blacks, Caucasians and Hispanics.

## Methods

### Participants and simple Plex assay

Subjects were enrolled in the study Prospective Collection of Samples for Research according to an IRB approved by Schulman Associates IRB (IRB # 201301461) for Bioreclamation*IVT*. Samples used in this study were purchased from Bioreclamation*IVT*. Concentration of ASC and IL-18 in the serum of healthy males (20 to 45 y/o and older than 45) as well as in Blacks, Caucasians and Hispanics was analyzed as described in [6] using the Ella System (Protein System). The Simple Plex assay was analyzed by the Simple Plex Explorer software. Results are the mean of samples run in triplicates. Sample size in this section of the study for ASC was 17 for the 20 to 45 y/o group and 40 for the > 45 y/o group; for IL-18: 19 for the 20 to 45 y/o group and 39 for the > 45 y/o group. For ASC: 31 Blacks, 15 Caucasians and 8 Hispanics. For IL-18: 35 Blacks, 16 Caucasians and 8 Hispanics.

### Animals

Male BALB/c mice were obtained from the National Institute on Aging (NIA) at 3 and 18 months old. All animal procedures were approved by the Animal Care and Use Committee of the University of Miami (protocol number 15–126). Animal procedures were carried according to the Guide for the Care and Use of Laboratory Animals published by the U.S. Public Health. Sample size in this section of the study consisted of 5 mice per group (5, 3-month-old mice and 5, 18-month-old mice). Studies were replicated 4 times.

### Isolation of cytosolic and mitochondrial fractions

Brains were removed from 3 and 18-month-old mice, and the cortex and hippocampus were dissected. Then protein was extracted using the Mitochondria Isolation kit for Tissue (ThermoFisher Scientific) according to manufacturer’s instructions. Briefly, tissue was homogenized on BSA/Reagent A solution. Mitochondria Isolation Reagent C was then added, and the preparation was centrifuged at 700 xg for 10 min at 4 °C. The supernatant was then further centrifuged at 3,000 xg for 15 min at 4 °C. The resulting supernatant corresponded to the cytosolic fraction. The mitochondrial pellet was then washed with Wash Buffer and centrifuged at 12,000 xg for 5 min. The supernatant was discarded and the mitochondrial fraction further processed for immunoblot analysis.

### Immunoblotting

Protein levels of inflammasome signaling proteins were determined in the cytosolic and mitochondrial fractions as described in [7]. Briefly, protein lysates were resolved in 10–20% Criterion TGX Stain-Free precasted gels (Bio-Rad), using antibodies (1:1000 dilution) to NLRC4 (Novus Biologicals, cat# NBP2–41124), caspase-1 (Novus Biologicals, cat# NB100–56565), caspase-11 (Novus Biologicals, cat# MAB8648), ASC (Santa Cruz, cat# sc-271054), IL-1β (Cell Signaling, cat# 12242S), IL-18 (Abcam, cat# ab71495), HSP60 (Cell Signaling, cat#12165) and β-actin (Sigma Aldric, cat# A5441). Quantification of band densities was done using the UN-SCAN-IT gel 6.3 Software (Silk Scientific Corporation). Chemilluminescence substrate (LumiGlo, Cell Signaling) was imaged with the ChemiDoc Touch Imaging System (BioRad).

### Pyroptosome isolation assay

Pyroptosome isolation was performed as described in [8]. Accordingly, mice cortical and hippocampal tissue lysates were filtered through a 50-μm low-binding polyvinylidene diflourride (PVDF) membrane (Milipore) and centrifuged at 2,700 xg for 8 min. The pellet was then resuspended in 3[(3-cholamidopropyl) dimethylammonio]-propanesulfonic acid (CHAPS) buffer with protease inhibitor cocktail. The pyroptosome was then pelleted by centrifugation at 2,700 xg for 8 min and re-suspended and incubated with CHAPS buffer with 2 mM of disuccinimidyl substrate (DSS) for 30 min at room temperature to cross-link ASC oligomers. Protein lysates were then analyzed by immunoblotting using antibodies to ASC and Gasdermin-D (GSDM-D, Novus Biologicals, cat# NBP2–33422).

### Immunocytochemistry

Human fibroblasts were obtained from the NIA Aging Cell Repository (Coriell Institute for Medical Research) from an individual who donated his cells at the ages of 49, 52 and 64 y/o). Cells were then grown in culture, and immunocytochemical analysis was carried by growing the cells on 12 well-plates. Cells were then fixed with 10% formaldehyde and permeabilized with blocking buffer containing 5% normal goat serum and 0.1% Triton in PBS. Cells were incubated with a primary antibody against caspase-1 (Novus Biologicals) diluted 1:200 in blocking buffer followed by fluorescently labeled Alexa-Fluor 488 (ThermoFisher Scientific) secondary antibody diluted 1:200 in blocking buffer and Phalloidin (Cytoskeleton). Images were obtained with an EVOS FL Auto 2 Imaging System (FisherScientific). Secondary antibody alone was used as a negative control.

### LDH release assay

To assess cell viability of fibroblasts following oxidative stress, human fibroblasts corresponding to the ages of 49 and 52 y/o were grown in culture and exposed to 25 μM hydrogen peroxide for 30 min. Then, the CytoTox 96 Non-Radioactive Cytotoxicity Assay (Promega, Madison, WI) was used according to manufacturer’s instructions to evaluate the number of lysed cells as described in [6]. Another group of cells (52 years-old) was exposed to 25 μM hydrogen peroxide and 10 μg/ml of the caspase-1 inhibitor Ac-YVAD-CMK (Invivogen) prior to LDH release analysis. Ac-YVAD-CMK is a cell permeable, irreversible, selective inhibitor that prevents the cleavage of caspase-1, thus preventing its activation. Studies were replicated 5 times for all groups.

### Cellular ROS determination

The Cellular ROS Detection Assay kit was used to determine the levels of ROS produced following challenge of fibroblasts (52 y/o) with hydrogen peroxide (25 μM) for 30 min with and without inhibition of the inflammasome with Ac-YVAD-CMK (10 μg/ml) according to manufacturer’s instructions. Briefly, cells were grown in a 96 well plate and stained with 2′,7′-Dichlorodihydrofluorescein diacetate (DCDFA) for 45 min at 37 °C. Cells were then washed and exposed to hydrogen peroxide and/or Ac-YVAD-CMK as described. Fluorescence was then measured with an excitation of 485 nm and an emission of 535 nm in a microplate reader. Studies were replicated 9 times for both groups.

### Statistical analyses

Data are shown as mean ± standard error of the mean (SEM) with a p of less than 0.05 considered as significant in all statistical tests. Statistical comparisons between groups were done using a student’s t-test or a one-way ANOVA followed by a Kruskall-Wallis test, as appropriate.

## Results

### ASC and IL-18 are elevated in the serum of people over 45 y/o

**Fig. 1:**
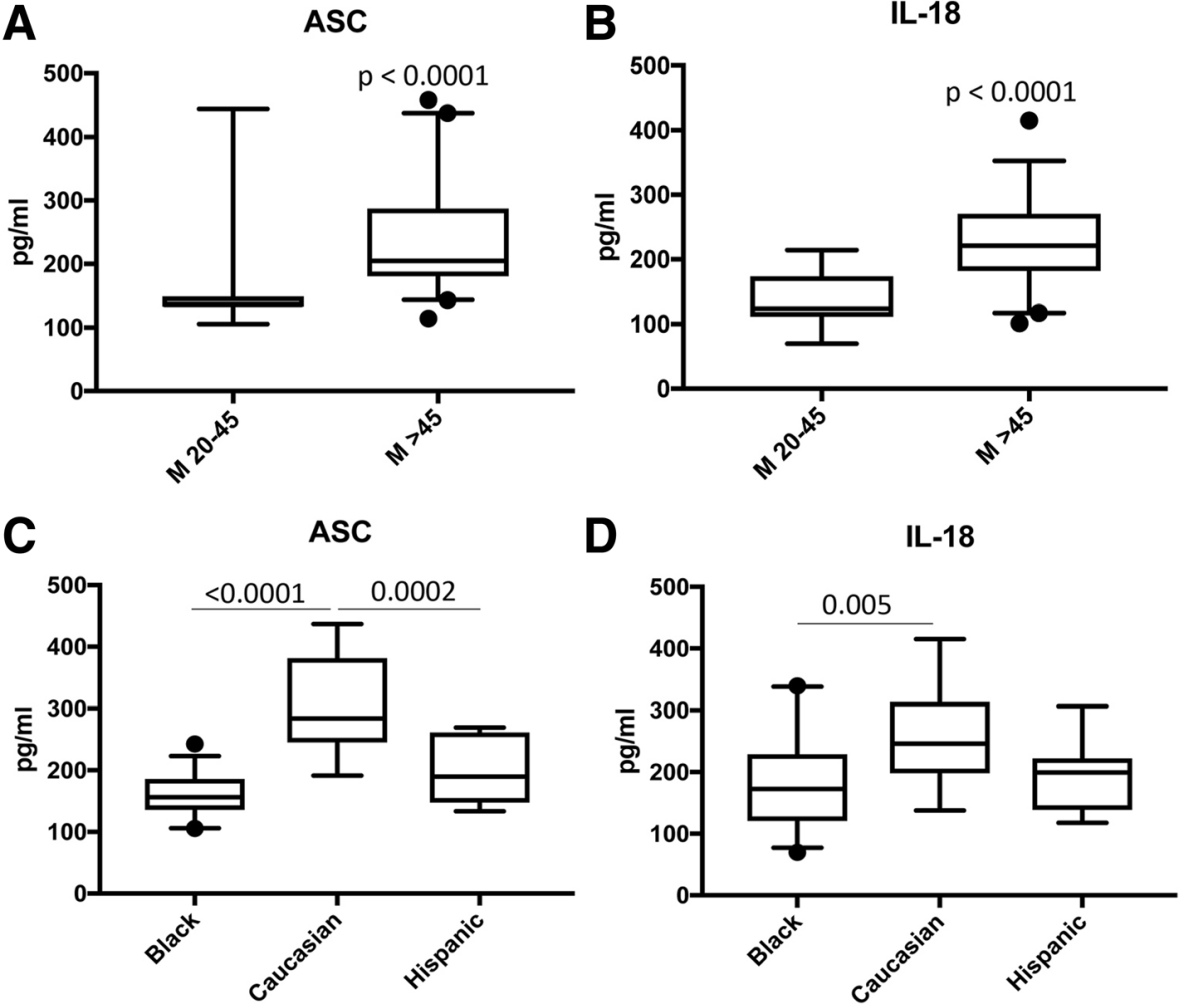
ASC and IL-18 proteins are elevated in the serum of males over 45 y/o and in Caucasians when compared to Blacks and Hispanics**:** Protein levels in pg/ml of ASC (**a**) and IL-18 (**b**) in serum samples from healthy donors. Samples were then divided into Blacks, Caucasians and Hispanics (**c** and **d**). *p*-value of significance is shown above each box plot. Box and whiskers are shown for the 5th and 95th percentile. ASC: *N* = 17, 20 to 45 y/o and *N* = 40, > 45 y/o. IL-18: *N* = 19, 20 to 45 y/o and *N* = 39, > 45 y/o. ASC: *N* = 31 Black, *N* = 15 Caucasian and *N* = 8 Hispanic. IL-18: *N* = 35 Black, *N* = 16 Caucasian and N = 8 Hispanic

To determine the levels of the inflammasome proteins ASC and IL-18 in the serum of healthy donors, we analyzed serum samples of healthy male volunteers using the Ella Simple Plex System and divided the samples into two groups, males 20 to 45 y/o and males older than 45 y/o. Accordingly, we determined that the levels of ASC (Fig 1a) and IL-18 (Fig 1b) in these healthy volunteers were greater in the older cohort when compared to the younger cohort. Thus, indicating that inflammasome protein expression increases with increased age even in healthy subjects.

### ASC protein levels are higher in the serum of Caucasians when compared to blacks and Hispanics

To determine if there is a difference in the protein levels of ASC and IL-18 in healthy donors across ethnicities, we stratified the group of samples from male donors into Blacks, Caucasians and Hispanics and determined the protein levels of ASC and IL-18 in serum. The samples from healthy Caucasians presented higher levels of ASC when compared to Blacks and Hispanics (Fig 1c). In regards to IL-18, the protein levels were higher in Caucasians than in Blacks; however, no statistical significance was found between Caucasians and Hispanics (Fig 1d). These findings imply a difference in ASC and IL-18 protein levels across ethnicities within healthy individuals.

### NLRC4 inflammasome signaling proteins are elevated in the cortex and hippocampus of aged mice

**Fig. 2:**
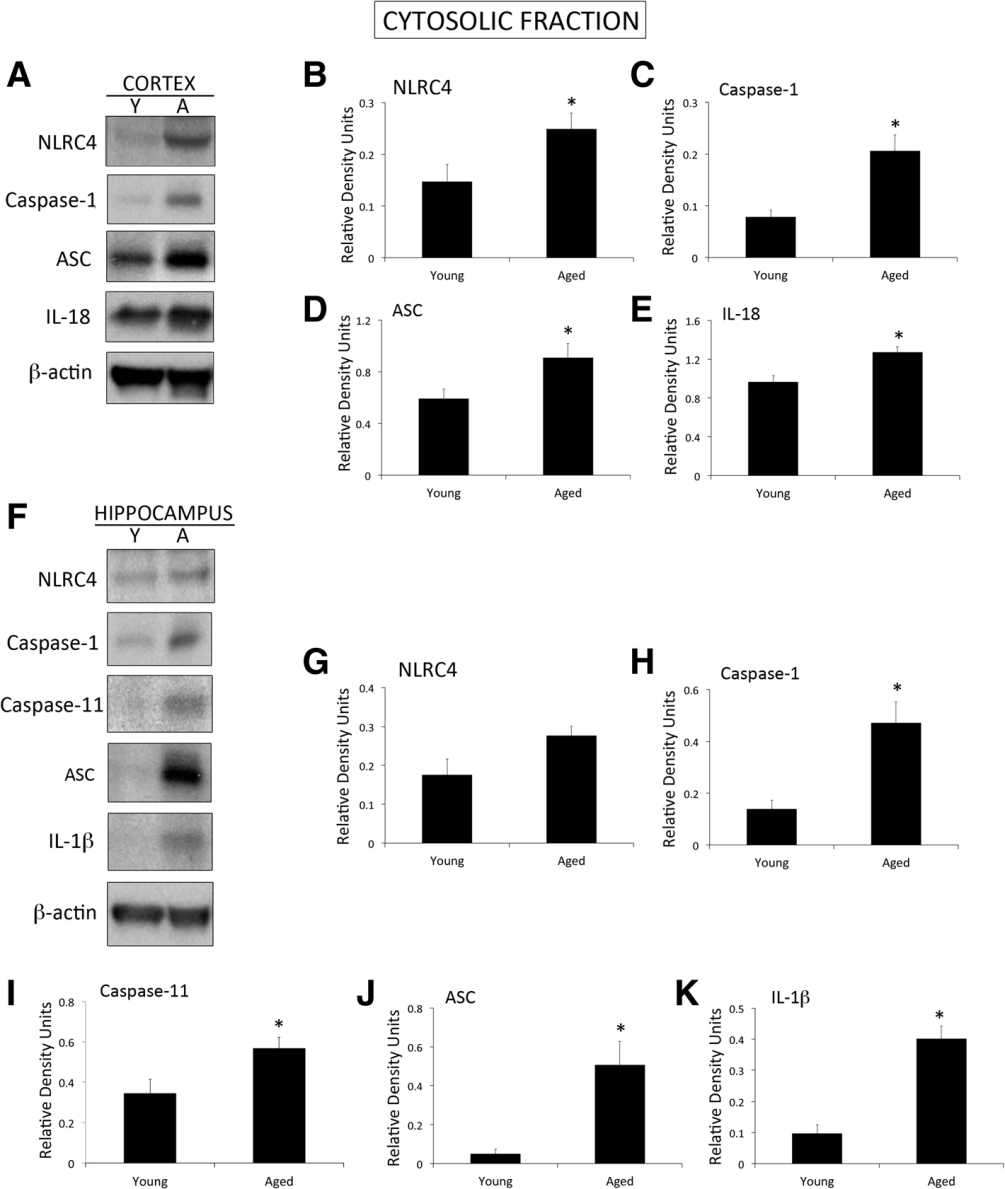
Inflammasome proteins are elevated in the cytosolic fraction of aged mice: **a** Representative image of immunoblot analyses of inflammasome proteins in the cytosolic fraction of the cortex of young (Y) and aged (A) mice. Quantification of immunoblot analysis of NLRC4 (**b**), caspase-1 (**c**), ASC (**d**), IL-18 (**e**) in the cortex. **a** Representative image of immunoblot analyses of inflammasome proteins in the cytosolic fraction of the hippocampus of young (Y) and aged (A) mice. Quantification of immunoblot analysis of NLRC4 (**g**), caspase-1 (**h**), caspase-11 (**i**), ASC (**j**), IL-1β (**k**) in the hippocampus. Data presented as mean+/-SEM. *N* = 5 per group. *p < 0.05

To determine whether aging increases inflammasome protein expression in the cortex and hippocampus of aged mice, we obtained 3 and 18-month-old mice from the NIA and dissected their cortices (Fig 2a) and hippocampi (Fig 2f) to then isolate the cytosolic fraction to determine the protein levels of the inflammasome signaling proteins NLRC4 (Fig 2b and g), caspase-1 (Fig 2c and h), ASC (Fig 2d and j), IL-18 (Fig 2e), caspase-11 (Fig 2i) and IL-1β (Fig 2k). Our data indicate that NLRC4, caspase-1, ASC and IL-18 were elevated in the cytosol of cortical cells corresponding to the 18-month-old mice, and that caspase-1, caspase-11, ASC and IL-1β were elevated in the cytosolic fraction of hippocampal cells. These findings are consistent with our previous report showing that inflammasome proteins are elevated in the aging brain of rodents [4] and expand our knowledge regarding the cytosolic distribution of these proteins.

### ASC is elevated in the mitochondrial fraction in the cortex and hippocampus of aged mice

**Fig. 3:**
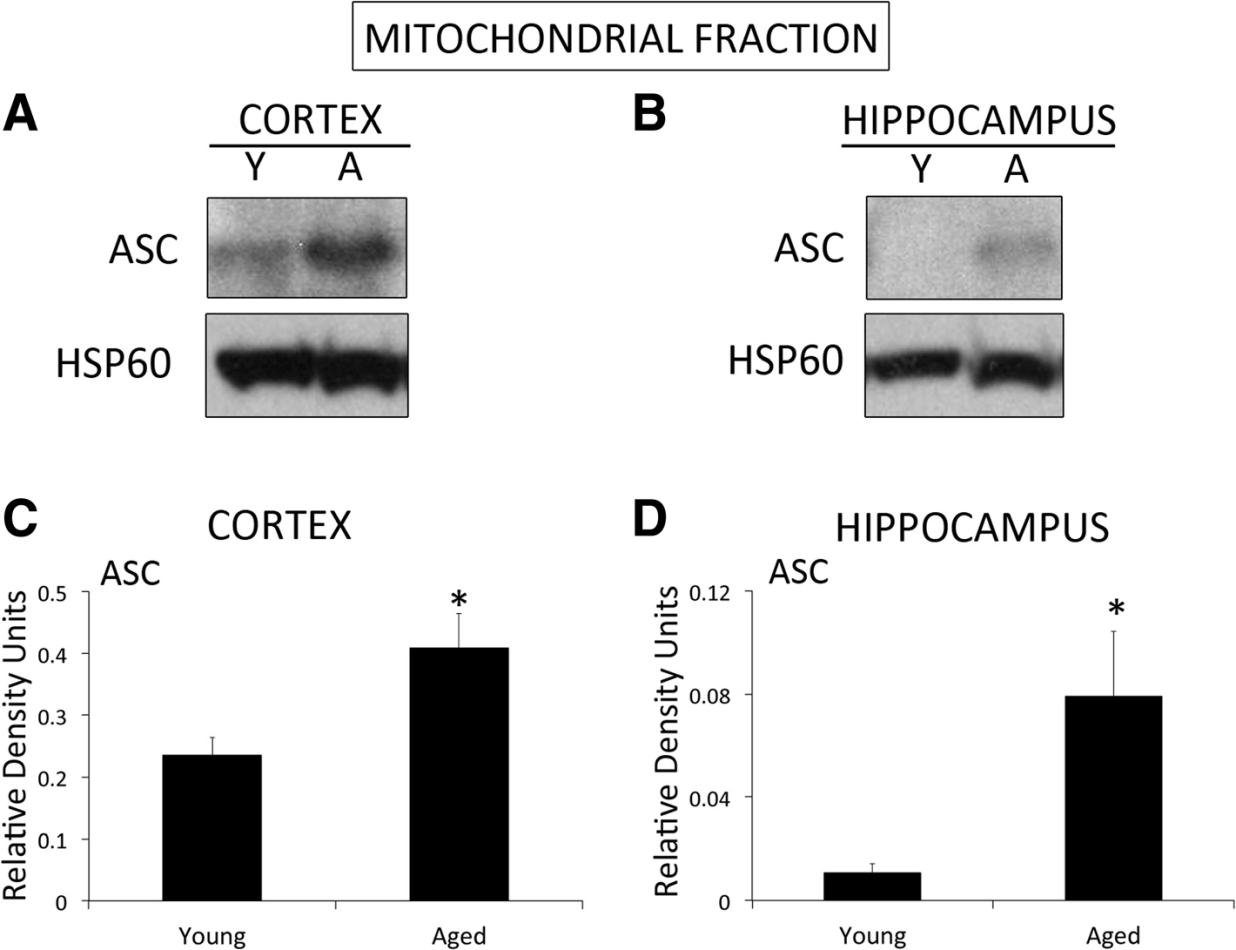
Inflammasome proteins are elevated in the mitochondrial fraction of aged mice: **a** Representative image of immunoblot analysis of ASC in the mitochondrial fraction of the cortex (**a**) and hippocampus (**b**) of young (Y) and aged (A) mice. Quantification of immunoblot analysis of ASC in the cortex (**c**) and hippocampus (**d**). Data presented as mean+/-SEM. N = 5 per group. * *p* < 0.05

Mitochondria have been shown to play a key role in Inflammasome activation [9, 10], we thus decided to determine if inflammasome signaling proteins were present in mitochondria and found that ASC is present at higher levels in the mitochondria of aged mice in both, the cortex (Fig 3a) and hippocampus (Fig 3b), when compared to young mice (Fig 3c and d). Thus, suggesting a role for ASC in mitochondria.

### Pyroptosis is increased in the cortex and hippocampus of aged mice when compared to young

**Fig. 4:**
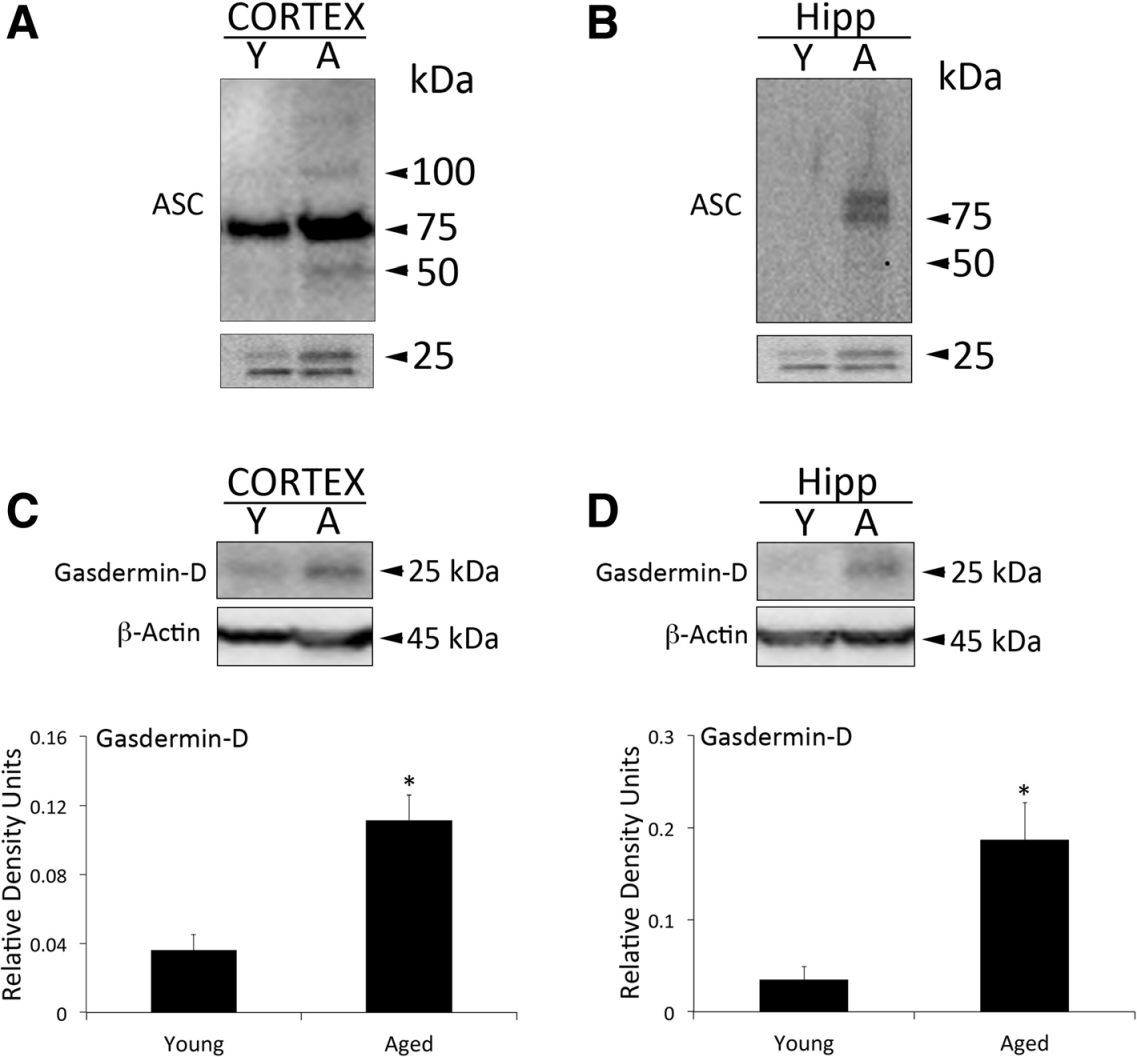
Pyroptosome formation in the cortex and hippocampus of aged mice: Aging induces laddering of ASC in cortex (**a**) and hippocampus (**b**) of mice, indicating formation of the pyroptosome, an oligomerization of ASC that leads to pyroptosis. Representative immunoblot and quantification of gasdermin-D in the cortex (**c**) and hippocampus (**d**) of aged mice when compared to young. Gasdermin-D is significantly elevated in the cortex and hippocampus of aged mice. Data presented as mean+/-SEM. N = 5 per group. * *p* < 0.05

Since ASC has been shown to oligomerize in the inflammasome-mediated cell death mechanism of pyroptosis, we decided to compare the levels of ASC oligomerization in the cortex (Fig 4a) and hippocampus (Fig 4b) of aged and young mice, and found that the ASC oligomerization was more evident in the cortex than the hippocampus, and that this oligomerization was greater in aged mice when compared to young. Moreover, we then further analyzed the protein levels of the substrate of pyroptosis gasdermin-D [8] and found that the cleaved fragments of gasdermin-D were expressed at higher levels in the cortex (Fig 4c) and hippocampus (Fig 4d) of aged mice when compared to young. These findings suggest that in the aging brain, there is a naturally occurring process of cell death that is mediated in part by the inflammasome.

### Caspase-1 and ASC protein levels increase over time in human fibroblasts

**Fig. 5:**
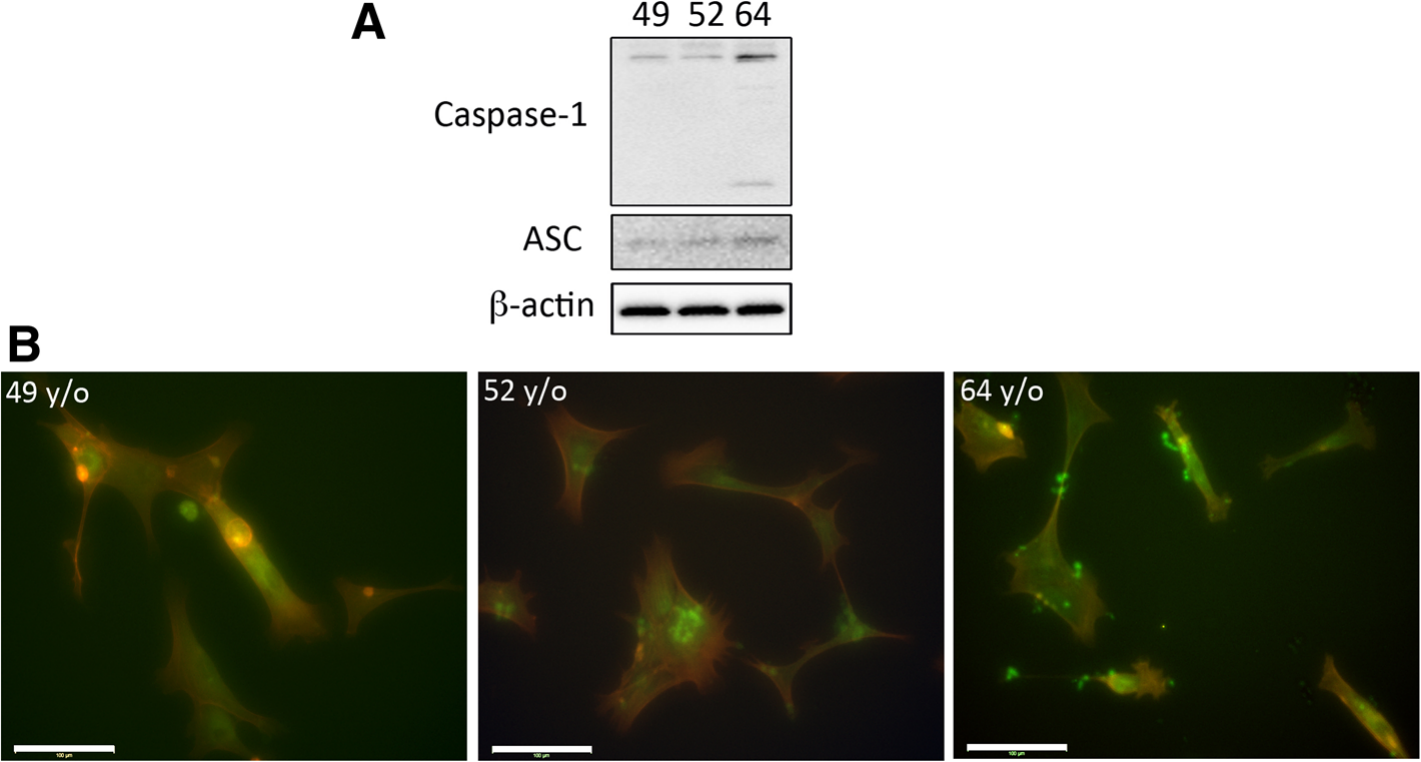
Caspase-1 expression increases with age in human fibroblasts: **a** Immunoblot analysis of caspase-1 and ASC in human fibroblasts obtained from a donor at the ages of 49, 52 and 64 y/o. **b** Immunocytochemistry image of human fibroblasts stained for caspase-1 (green) and Phalloidin (red) from a donor at three different ages (49, 52 and 64 y/o). Bar graph**: 100** μm

To further study the effects of aging on inflammasome protein expression, we obtained human fibroblasts from a subject who donated his cells at three different ages: 49, 52 and 64 y/o. Interestingly, in the 64 y/o cells, we were able to detect higher protein levels of caspase-1 and ASC (Fig 5a). This finding was consistent with increased immunoreactivity of caspase-1 (green) in Phalloidin-stained fibroblasts (red) as detected by immunocytochemistry followed by fluorescent microscopy (Fig 5b). These data suggest that in aging cells there is a naturally occurring increase in inflammasome protein expression.

### Oxidative stress increases LDH release in fibroblasts from a donor at 52 y/o when compared to 49 y/o

**Fig. 6:**
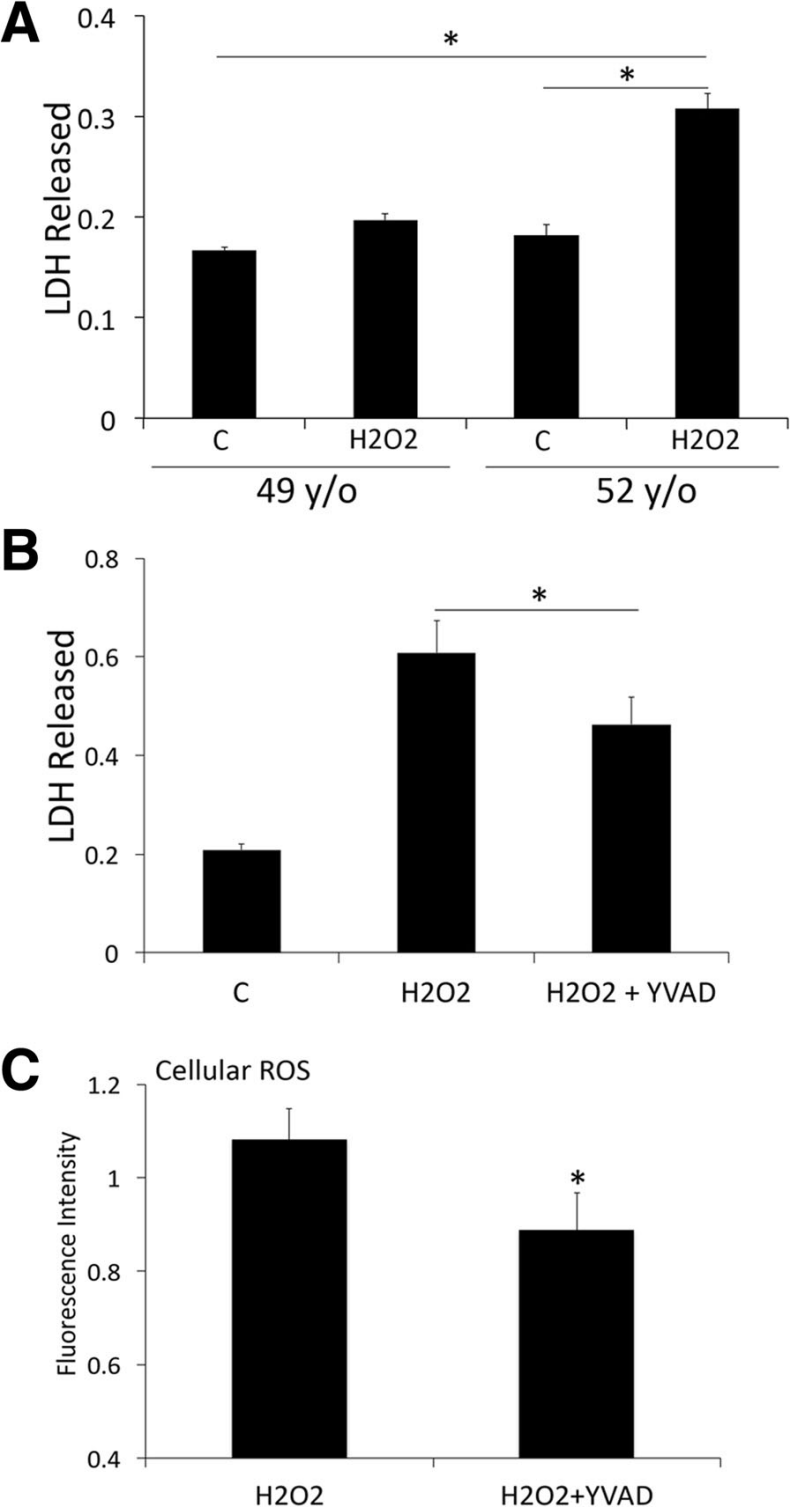
The inflammasome mediates oxidative stress resulting in LDH release and production of ROS: Bar graph of LDH released by human fibroblasts (49 y/o) after exposure to hydrogen peroxide (25 μM H2O2) for 30 min (**a**) and in 52 y/o cells after inhibition of caspase-1 with YVAD (**b**). Cellular ROS production after challenge of 52 y/o fibroblasts with 25 μM H2O2 for 30 min and inhibition of caspase-1 with YVAD (**c**)

Oxidative stress is a natural consequence and contributor of the aging process [11], thus we then proceeded to expose 49 and 52 y/o fibroblasts to 25 μM of hydrogen peroxide for 30 min and then measured the amount of LDH released following oxidative stress. Interestingly, the levels of LDH released were higher in the 52 y/o cells when compared to control cells of the same age that were not exposed to hydrogen peroxide. However, there was no statistical difference between control and hydrogen peroxide-exposed 49 y/o cells (Fig 6a), suggesting that older cells are more prone to damage following oxidative stress than younger cells.

### Inflammasome inhibition decreases LDH release and ROS formation in human aged fibroblasts

We then exposed the 52 y/o cells to hydrogen peroxide and the caspase-1 inhibitor **Ac-YVAD-CMK**. Importantly, inhibition of caspase-1 after oxidative stress decreased the levels of LDH released (Fig 6b**)** and the levels of cellular ROS produced (Fig 6c). Taken together, these data indicate that inhibition of the inflammasome in the aging process has the potential to treat the cellular damage associated with oxidative stress.

## Discussion

Aging is a common denominator in several neurodegenerative diseases such as Parkinson’s disease or Alzheimer’s disease. Inflammation is a major factor in a myriad of diseases, and inflammaging is part of the normal process in an individual’s life cycle. It has been previously shown that the inflammasome is a key contributor to the innate immune response seen in the aging population [4, 12–19].

In this study, we show that the protein levels of ASC and IL-18 in serum are higher in males over 45 y/o than in the 20 to 45 y/o group. When we divided samples from these subjects into Blacks, Caucasians and Hispanics we found that ASC was higher in Caucasians than in the other two groups, and that IL-18 was higher in Caucasians than in Blacks. However, there was no significant difference between Caucasians and Hispanics in regards to IL-18. Moreover, we attempted to measure the levels of caspase-1 and IL-1β in these samples, but the levels of these proteins were so low in samples from healthy donors, specially the younger cohort, that the amounts of protein detected were below the level of detection of the assay (data not shown). We have recently shown that ASC is elevated in the serum of patients with multiple sclerosis [20]. However, in this study we present data showing that the levels of ASC differ between these age groups and ethnicities. Thus, highlighting the importance of considering age [21–26] and ethnicity when studying the innate immune response [21, 27–29].

We have previously shown that inflammasome signaling proteins are elevated in the brain of aged rats when compared to young [4]. In this study, we extend these findings to show that NLRC4, caspase-1, ASC and IL-18 are elevated in the cytosolic fraction of cortical lysates in the aged brain when compared to young. Similarly, NLRC4, caspase-1, caspase-11, ASC and IL-1β were elevated in the hippocampus. These data suggest a role for the NLRC4 inflammasome in the innate immune response of the aging brain.

We have previously shown that the inflammasome-mediated cell death mechanism of pyroptosis occurs in cortical neurons [30]. Here we show that pyroptosome formation as determined by oligomerization of the inflammasome adaptor protein ASC is evident in cortical and hippocampal lysates of the brain of aged mice when compared to young. This was consistent with increased levels of the recently identified substrate of pyroptosis gasdermin-D [31–34]. These findings suggest that in the aging brain, there is a natural process of cell death that is in part mediated by the inflammasome, which is consistent with previous findings indicating that indeed in the aging brain there is a cell death process [35–39]. Taken together, this highlights the potential for inflammasome-mediated naturally occurring cell death associated with inflammaging as a precursor to the development of neurodegenerative diseases like as Parkinson’s disease and Alzheimer’s disease.

Most degenerative conditions are characterized by low-grade inflammation [40]. Moreover, mitochondrial dysfunction is at the core of many diseases in addition to aging [41]. The brain consumes about 20 to 25% of the body’s total energy [42]. Thus it is an organ that undergoes major metabolic demands. Most of this energy is spent in the process of neurotransmission and is spent by mitochondria [43]. As we age, mitochondrial electron transport chain function declines, as the production of free radicals increases [44]. In the CNS neurons are highly aerobic, whereas astrocytes rely more on glycolysis as their energy source. Thus, the neuronal-astrocyte interaction is important when considering energetics. Accordingly, neurotransmission activates glycolysis and lactate production in astrocytes, and this lactate in turn triggers oxidative phosphorylation [45]. Moreover, in the aging brain, within mitochondria, there is a decline in the activity of complexes I (NADH) and IV (cytochrome oxidase) [46]. In the CNS, with increased age, oxidative stress markers increase as well [47]; thus, there is an important role for mitochondria in the process of aging.

In this study, we show that ASC is elevated in the mitochondrial fraction of the cortex and hippocampus of aged mice when compared to young, consistent with previous reports indicating a role for mitochondria in inflammasome signaling [9, 10]. To further study the role of oxidative stress and the aging process as it pertains to inflammasome signaling, we obtained fibroblasts from a subject who donated his cells at three different ages (49, 52 and 64 y/o) and discovered that caspase-1 and ASC protein levels were higher at the oldest time-point analyzed than at the other two younger time-points. Moreover, the 52 y/o cells were more prone to cell death (as determined by LDH release) when subjected to oxidative stress when compared to the 49 y/o cells. Thus, highlighting the vulnerability of cells to oxidative stress due to the aging process. Importantly, when the inflammasome was inhibited with the caspase-1 inhibitor Ac-YVAD-CMK in these cells following oxidative stress, the amount of LDH released and the amount of cellular ROS produced was decreased. Therefore, these findings indicate that inflammasome inhibition can ameliorate the effects of oxidative stress (reduced ROS production) and decreased cell damaged (reduced LDH release) associated with aging.

A limitation of this study is that here we have used cells across different ages from only one donor; thus, further studies need to address whether this effect of increased inflammasome signaling, increased LDH release and increased production of ROS occurs in cells from other donors as well. Moreover, in this study we have only analyzed serum samples from male donors. Therefore, future studies need to also determine the protein changes of inflammasome signaling proteins in females of different ages.

In conclusion, this is the first report to show that pyroptotic cell death occurs in the aging brain and that the inflammasome can be a viable target to decrease the oxidative stress that occurs as a result of aging. In addition, this is the first report to show the protein levels of two key inflammasome signaling proteins ASC and IL-18, in two cohorts of subjects (20 to 45 y/o and older than 45 y/o) as well as in Blacks, Caucasians and Hispanics. Current studies are evaluating more closely the role that different inflammasome signaling proteins play in mitochondrial function and oxidative stress as well as in cognitive performance as a result of aging.

Taken together, in this study we present different aspects on the role of the inflammasome in inflammaging. First, we report the protein expression profile of ASC and IL-18 in healthy individuals across different ages and different ethnicities, and second, we have expanded on our previous findings regarding the role of the inflammasome on brain inflammaging by identifying pyroptosis as a contributor to cell death in the aging brain, as well as the role of oxidative stress on inflammasome activation at different ages.
